# Evaluation of candidate reference genes for quantitative real-time PCR analysis in a male rat model of dietary iron deficiency

**DOI:** 10.1186/s12263-021-00698-0

**Published:** 2021-10-02

**Authors:** Joanna L. Fiddler, Stephen L. Clarke

**Affiliations:** 1 Division of Nutritional Sciences, Cornell University, Ithaca, NY 14850-6301 USA; 2 Department of Nutritional Sciences, Oklahoma State University, Stillwater, OK 74078 USA

**Keywords:** Anemia, housekeeping genes, normalization genes, mRNA expression, quantitative real-time PCR

## Abstract

**Background:**

Quantitative real-time polymerase chain reaction (qPCR) is a reliable and efficient method for quantitation of gene expression. Due to the increased use of qPCR in examining nutrient-gene interactions, it is important to examine, develop, and utilize standardized approaches for data analyses and interpretation. A common method used to normalize expression data involves the use of reference genes (RG) to determine relative mRNA abundance. When calculating the relative abundance, the selection of RG can influence experimental results and has the potential to skew data interpretation. Although common RG may be used for normalization, often little consideration is given to the suitability of RG selection for an experimental condition or between various tissue or cell types. In the current study, we examined the stability of gene expression using BestKeeper, comparative delta quantitation cycle, NormFinder, and RefFinder in a variety of tissues obtained from iron-deficient and pair-fed iron-replete rats to determine the optimal selection among ten candidate RG.

**Results:**

Our results suggest that several commonly used RG (e.g., *Actb* and *Gapdh*) exhibit less stability compared to other candidate RG (e.g., *Rpl19* and *Rps29*) in both iron-deficient and iron-replete pair-fed conditions. For all evaluated RG, *Tfrc* expression significantly increased in iron-deficient animal livers compared to the iron-replete pair-fed controls; however, the relative induction varied nearly 4-fold between the most suitable (*Rpl19*) and least suitable (*Gapdh*) RG.

**Conclusion:**

These results indicate the selection and use of RG should be empirically determined and RG selection may vary across experimental conditions and biological tissues.

## Background

Iron is an essential nutrient and is involved in many mammalian processes including DNA synthesis, erythropoiesis, ATP production, and oxygen transport [1, 2]. In humans, iron deficiency (ID) remains the most common single nutrient deficiency and affects approximately 25% of the world’s population or 1.62 billion people according to the World Health Organization [3]. Due to its importance in biological functions, inadequate levels of iron lead to microcytic anemia, diminished cognitive development, and decreased ATP production [1, 4].

A variety of biomarkers and methodologies exist to investigate iron status; for example, measuring serum ferritin and transferrin saturation are common practices and often employed together to enhance the detection of systemic ID [5]. To investigate the iron content of biological tissues, inductively coupled plasma mass spectrometry is a useful strategy due to its low detection limits [6]. In many instances, however, indirect measures are needed to further understand iron homeostasis. In these instances, the addition of immunoblotting, quantitative real-time PCR (qPCR), and iron regulatory protein (IRP) RNA-binding assays can be utilized to determine the abundance of proteins such as ferritin and transferrin receptor [7], the gene expression of mRNA encoding proteins such as transferrin receptor or hepcidin [8], and IRP-binding activity, respectively [9]. Of these approaches, qPCR has become the gold standard for evaluating gene expression due to its sensitivity, accuracy, and simplicity [10, 11]. Therefore, fully understanding this technique and standardizing the methods, along with analyzing and interpreting qPCR results, are of great importance.

To compare differences in gene expression (i.e., mRNA abundance) between experimental groups, qPCR is used by applying Kary Mullis’ novel method of amplifying DNA and using probe-based chemistries [11–13]. Following exposure to experimental conditions, there are 4 major steps to successfully complete qPCR: (1) harvest quality RNA from experimental groups, (2) reverse transcribe RNA templates into complementary DNA (cDNA), (3) amplify cDNA with probe-based chemistries by qPCR, and (4) quantify relative mRNA abundance. First, it is essential that RNA integrity is maintained during isolation and purification as poor quality RNA may compromise experimental results [14]. Second, since qPCR amplifies only DNA by taking advantage of DNA polymerases, the quality RNA must be reverse transcribed into cDNA by the enzyme reverse transcriptase [11]. The third step, amplification of the cDNA, utilizes fluorescence-based molecules that bind to DNA and fluoresce following excitation. As each qPCR cycle is repeated, new copies of the cDNA template are generated. Thus, the fluorescence signal is directly proportional to the abundance of DNA. Finally, to quantify relative mRNA abundance, it is important to control for sample-to-sample variation by one of two strategies, the standard curve method or the comparative CT method. Both methods amplify a target gene and a control gene. The standard curve method expresses the relative mRNA abundance to the standard curve of the calibrator (i.e., untreated sample) and the comparative CT method normalizes the threshold cycle values of the target gene to the control gene before comparing experimental groups.

The normalization process accounts for discrepancies in RNA isolation, reverse transcription, and qPCR [15, 16]. Normalization utilizes invariant control genes that are typically referred to as “housekeeping” or “reference” genes (RG) [17]. Ideally, RG have minimal variation in tissue or cell type and under different experimental conditions; thus, RG are considered stable. Interestingly, many RG have been reported to be regulated by experimental conditions or tissue type [18] and subsequently influence gene expression interpretation [15].

To date, there are many studies on RG selection for a number of animal and cell models [19–22]; however, there is limited data regarding RG selection in animal models of dietary conditions. Consequently, the absence of a systematic approach to RG selection makes gene expression data potentially difficult to interpret and compare between studies, and therefore less reliable. For instance, Suzuki et al. [23] reported *Gapdh* and *Actb* were used as RG in more than 60% of articles they reviewed in high-impact journals. While these RG may have been appropriate in those studies, both are affected by hypoxia and cell cycle maturation [24, 25], and tissue type [26]. Furthermore, a commonly used iron-chelating reagent, desferrioxamine, is considered a hypoxia-mimetic which may also regulate these RG. Some progress has been made in terms of RG selection in certain models, though the extent to which these results can be applied to all models remains unclear [16, 21]. The focus of this study was to examine RG stability in a weanling rat model of dietary ID and to determine appropriate RG for use in qPCR. Additionally, the extent to which these RG were responsive to dietary ID was assessed. We examined the stability of gene expression in ten commonly used RG in qPCR (*Actb*, *Gapdh*, *Hprt*, *Ppia*, *Rpl19*, *Rpl22*, *Rpl27*, *Rplp0*, *Rps29*, and *Tbp*) for their candidacy to be used when comparing iron-deficient and iron-replete pair-fed (PF) rat experimental conditions. RG stability was also determined for individual tissues including the gastrocnemius, heart, kidney, liver, lung, and spleen under the same experimental conditions. Using four RG computational programs (BestKeeper, comparative delta quantification cycle (∆Cq), NormFinder, and RefFinder), we analyzed the gene stability to predict the most suitable RG for studying the effects of dietary ID on the regulation of gene expression [2, 20, 22, 27].

## Results

Animal anthropometric data and iron status measurements throughout the study are published elsewhere [28]. In summary, the ID group exhibited greater than 50% reduction in hemoglobin, hematocrit, and serum iron levels compared to both the control (C) and PF groups. ID animals weighed ~ 20% less than the C group; therefore, to control for the total diet consumed, the PF group was fed an iron-sufficient diet to the level of the ID group’s daily consumption. Importantly, there were no differences in final body weight or rate of weight gain between PF and ID groups. These results are consistent with previous findings indicating that ID animals exhibit decreased food intake and lower body weight compared to C animals [29]. All RG analyses were made utilizing the PF group instead of the C group to account for any non-specific changes due to unequal food intake.

### BestKeeper analysis

BestKeeper software analysis ranks RG based on a pairwise correlation and then calculates the most suitable RG based on geometric means assessing crossing points (CP) or Cq. Among potential RG examined, if the criterion (SDCq value < 1.0) was met, RG were considered suitable for qPCR normalization [27]. Interestingly, when analyzing RG in both experimental groups (PF and ID) in individual tissue, all RG except one exhibited stability based on the criteria (data not shown). *Rplp0* failed to meet the criteria in heart tissue (SDCq = 1. 2). After analyzing each experimental group individually using an all tissue combined approach, BestKeeper analyses indicated high variation in RG expression in the PF group with only *Hprt* meeting the criteria (Table 1) and moderate variation in the ID group with five of the candidate genes *Hprt*, *Rps29*, *Tbp*, *Rpl19*, and *Rplp0* having an SDCq value < 1.0 (Table 2). Finally, when combining datasets from all tissues and both experimental groups to determine which RG exhibits the least amount of variability, *Hprt* and *Rpl19* displayed the most stability (Table 3). Interestingly, two commonly used RG in the rat model of ID and other nutrition models, *Actb* and *Gapdh*, exhibited poor stability with *Actb* having the least stability in all BestKeeper analyses [30–32].

**Table 1:** BestKeeper descriptive statistics and ranking of reference genes in pair-fed iron-replete animals in all tissues

	***Hprt***	***Rps29***	***Rplp0***	***Tbp***	***Rpl27***	***Rpl22***	***Gapdh***	***Actb***	***Ppia***	***Rpl19***
**Geo mean [CP]**	22.68	17.72	19.08	24.32	18.3	17.89	17.92	17.13	19.89	22.72
**CV**	0.02	0.06	0.06	0.05	0.06	0.06	0.07	0.07	0.06	0.06
**Min [CP]**	21.28	15.75	17.05	22.54	15.77	15.87	16.13	14.85	17.79	19.52
**Max [CP]**	24.66	20.73	22.36	26.45	21.15	20.38	20.65	19.9	25.65	25.29
**Std dev [± CP]**	0.51	1.06	1.08	1.11	1.13	1.14	1.17	1.22	1.24	1.37
**Min [*x*-fold]**	− 2.64	− 3.92	− 4.08	− 3.45	− 5.78	− 4.04	− 3.46	− 4.85	− 4.27	− 9.18
**Max [*x*-fold]**	3.94	8.05	9.71	4.37	7.21	5.64	6.63	6.85	54.44	5.92
**Std dev [±*x*-fold]**	1.42	2.08	2.11	2.16	2.19	2.2	2.25	2.33	2.37	2.59
**Ranking**	**1**	**2**	**3**	**4**	**5**	**6**	**7**	**8**	**9**	**10**

Geometric mean (CP), coefficient of variance (CV), and standard deviation (± CP) of the Cq values for putative reference genes. RG are ordered from left to right according to their SD_Cq value_. Reference genes with a SD_Cq_ value < 1.0 are considered to be an appropriate reference gene when assessing gene expression in the pair-fed animals. To determine the under- and over-expression of a reference gene relative to the gene’s geometric mean (*x*-fold), the min, max, and standard deviation are calculated by the BestKeeper software

**Table 2:** BestKeeper descriptive statistics and ranking of reference genes in iron-deficient animals in all tissues

	***Hprt***	***Rps29***	***Tbp***	***Rpl19***	***Rplp0***	***Ppia***	***Rpl27***	***Rpl22***	***Actb***	***Gapdh***
**Geo mean [CP]**	22.57	17.48	24.16	17.44	19.2	17.6	18.8	17.99	16.64	22.47
**CV**	0.03	0.05	0.04	0.05	0.05	0.06	0.06	0.06	0.07	0.07
**Min [CP]**	21.13	15.55	21.67	15.45	17.26	15.48	16.62	15.68	14.49	19.18
**Max [CP]**	24.39	18.96	25.7	18.51	20.59	19.05	20.78	19.59	18.16	25.85
**Std dev [± CP]**	0.64	0.89	0.91	0.93	0.94	1.01	1.06	1.08	1.14	1.48
**Min [*x*-fold]**	− 2.7	− 3.81	− 5.6	− 3.98	− 3.83	− 4.35	− 4.53	− 4.95	− 4.42	− 9.75
**Max [*x*-fold]**	3.55	2.79	2.91	2.11	2.62	2.73	3.95	3.02	2.88	10.4
**Std dev [±*x*-fold]**	1.56	1.86	1.88	1.9	1.92	2.01	2.09	2.12	2.2	2.8
**Ranking**	**1**	**2**	**3**	**4**	**5**	**6**	**7**	**8**	**9**	**10**

Geometric mean (CP), coefficient of variance (CV), and standard deviation (± CP) of the Cq values for putative reference genes. RG are ordered from left to right according to their SD_Cq value_. Reference genes with a SD_Cq_ value < 1.0 are considered to be an appropriate reference gene when assessing gene expression in the iron-deficient animals. To determine the under- and over-expression of a reference gene relative to the gene’s geometric mean (*x*-fold), the min, max, and standard deviation are calculated by the BestKeeper software

**Table 3:** BestKeeper descriptive statistics and ranking of reference genes in iron-replete and iron-deficient animals in all tissues

	***Hprt***	***Rpl19***	***Rps29***	***Tbp***	***Rpl27***	***Rplp0***	***Ppia***	***Rpl22***	***Actb***	***Gapdh***
**Geo mean [CP]**	22.62	17.58	17.69	24.24	18.94	19.55	17.76	18.15	16.89	22.60
**CV**	0.03	0.06	0.06	0.04	0.06	0.06	0.06	0.06	0.07	0.06
**Min [CP]**	21.13	15.45	15.55	21.67	16.62	17.26	15.48	15.68	14.49	19.18
**Max [CP]**	24.66	20.73	20.38	26.45	22.36	25.65	20.65	21.15	19.90	25.85
**Std dev [± CP]**	0.58	0.99	1.00	1.01	1.06	1.10	1.10	1.11	1.18	1.43
**Min [x-fold]**	− 2.81	− 4.39	− 4.39	− 5.94	− 5.00	− 4.88	− 4.86	− 5.52	− 5.26	− 10.66
**Max [*x*-fold]**	4.10	8.85	6.47	4.62	10.67	68.84	7.38	8.00	8.10	9.52
**Std dev [±*x*-fold]**	1.49	1.99	2.00	2.01	2.08	2.14	2.15	2.16	2.27	2.70
**Ranking**	**1**	**2**	**3**	**4**	**5**	**6**	**7**	**8**	**9**	**10**

Geometric mean (CP), coefficient of variance (CV), and standard deviation (± CP) of the Cq values for putative reference genes. RG are ordered from left to right according to their SD_Cq value_. Reference genes with a SD_Cq_ value < 1.0 are considered to be an appropriate reference gene when assessing gene expression in pair-fed and iron-deficient animals. To determine the under- and over-expression of a reference gene relative to the gene’s geometric mean (*x*-fold), the min, max, and standard deviation are calculated by the BestKeeper software

### Comparative ΔCq analysis

Gene expression levels were analyzed for stability using the comparative ΔCq method and standard deviations (SD) [22]. Pairwise comparisons were utilized to determine ΔCq of the relative gene expression within individual tissues and in all tissues combined. Mean ΔCq and SD were then averaged to interpret RG stability values for each experimental condition individually (PF and ID) and combined experimental conditions stability among all tissues. Similar to Silver et al.’s results, certain genes exhibited increased or decreased levels of deviation in ΔCq among all tissues and experimental condition analyses [22]. Those genes calculated to have the lowest mean SD were interpreted as having the most stability as a RG. After examining treatment conditions separately, *Rpl22* and *Hprt* exhibited the most stability in PF animals and *Rpl19* and *Ppia* exhibited the most stability in ID animals. Finally, when combining datasets from each tissue and both experimental groups to determine which RG exhibits the most stability, *Rpl19* and *Actb* had the lowest mean SD and therefore the most stability, while *Rplp0* and *Ppia* had the highest SD or least stability (Table 4).

**Table 4:** Comparative ΔCq evaluation and ranking of RG in pair-fed and iron-deficient animals in all tissues

Sample	Mean ΔCq	SD	Mean SD	Sample	Mean ΔCq	SD	Mean SD	Sample	Mean ΔCq	SD	Mean SD	Sample	Mean ΔCq	SD	Mean SD
*Rpl19 vs Actb*	− 0.700	0.280	0.400	*Tbp vs Actb*	− 7.410	0.250	0.440	*Rps29 vs Rpl27*	1.460	0.400	0.590	*Rplp0 vs Gapdh*	2.110	0.650	1.450
*Rpl19 vs Rpl27*	1.430	0.280	(1)	*Tbp vs Rpl27*	− 6.070	0.250	(4)	*Rps29 vs Rpl19*	0.030	0.420	(7)	*Rplp0 vs Rpl19*	− 1.930	0.660	(10)
*Rpl19 vs Rpl22*	0.630	0.290		*Tbp vs Hprt*	− 2.240	0.280		*Rps29 vs Ppia*	0.440	0.510		*Rplp0 vs Ppia*	− 1.590	0.720	
*Rpl19 vs Ppia*	0.340	0.310		*Tbp vs Ppia*	− 6.380	0.310		*Rps29 vs Rpl27*	0.740	0.550		*Rplp0 vs Act*	− 2.620	0.740	
*Rpl19 vs Tbp*	6.700	0.360		*Tbp vs Rpl19*	− 6.700	0.360		*Rps29 vs Actb*	− 0.590	0.600		*Rplp0 vs Hprt*	2.050	0.910	
*Rpl19 vs Rps29*	− 0.030	0.420		*Tbp vs Rpl27*	− 5.280	0.360		*Rps29 vs Tbp*	6.820	0.650		*Rplp0 vs Rps29*	− 2.650	2.080	
*Rpl19 vs Hprt*	4.460	0.510		*Tbp vs Rps29*	− 6.820	0.650		*Rps29 vs Hprt*	4.570	0.680		*Rplp0 vs Rpl27*	− 1.210	2.140	
*Rpl19 vs Gapdh*	4.030	0.540		*Tbp vs Gapdh*	− 2.680	0.660		*Rps29 vs Gapdh*	4.140	0.740		*Rplp0 vs Rpl22*	− 1.880	2.290	
*Rpl19 vs Rplp0*	1.930	0.660		*Tbp vs Rplp0*	− 4.790	0.840		*Rps29 vs Rplp0*	2.030	0.750		*Rplp0 vs Tbp*	3.990	2.850	
*Actb vs Tbp*	7.410	0.250	0.420	*Rpl22 vs Rpl27*	0.800	0.240	0.450	*Gapdh vs Rpl19*	− 4.030	0.540	0.630				
*Actb vs Rpl19*	0.700	0.280	(2)	*Rpl22 vs Tbp*	6.070	0.250	(5)	*Gapdh vs Actb*	− 4.730	0.560	(8)				
*Actb vs Ppia*	1.030	0.290		*Rpl22 vs Ppia*	− 0.310	0.290		*Gapdh vs Rpl27*	− 2.600	0.590					
*Actb vs Rpl22*	1.330	0.320		*Rpl22 vs Rpl19*	− 0.630	0.290		*Gapdh vs Ppia*	− 3.700	0.620					
*Actb vs Rpl27*	2.120	0.390		*Rpl22 vs Hprt*	3.830	0.390		*Gapdh vs Hprt*	0.430	0.630					
*Actb vs Hprt*	5.160	0.410		*Rpl22 vs Actb*	− 0.870	0.460		*Gapdh vs Rplp0*	− 2.110	0.650					
*Actb vs Gapdh*	4.730	0.560		*Rpl22 vs Rps29*	− 0.740	0.550		*Gapdh vs Rpl22*	− 3.400	0.650					
*Actb vs Rps29*	0.590	0.600		*Rpl22 vs Gapdh*	3.400	0.650		*Gapdh vs Tbp*	2.680	0.660					
*Actb vs Rplp0*	2.620	0.740		*Rpl22 vs Rplp0*	1.750	0.910		*Gapdh vs Rps29*	− 4.140	0.740					
*Rpl22 vs Rpl27*	− 0.800	0.240	0.430	*Hprt vs Tbp*	2.240	0.280	0.530	*Ppia vs Actb*	− 1.030	0.290	1.180				
*Rpl27 vs Rpl19*	− 1.430	0.280	(3)	*Hprt vs Rpl22*	− 3.830	0.390	(6)	*Ppia vs Rpl19*	− 0.340	0.310	(9)				
*Rpl27 vs Tbp*	5.280	0.360		*Hprt vs Ppia*	− 4.140	0.420		*Ppia vs Hprt*	4.140	0.420					
*Rpl27 vs Ppia*	− 1.090	0.370		*Hprt vs Actb*	− 5.170	0.490		*Ppia vs Gapdh*	3.700	0.620					
*Rpl27 vs Actb*	− 2.120	0.390		*Hprt vs Rpl27*	− 3.030	0.500		*Ppia vs Rplp0*	1.570	0.720					
*Rpl27 vs Rps29*	− 1.460	0.400		*Hprt vs Rpl19*	− 4.460	0.510		*Ppia vs Rpl22*	− 0.290	1.870					
*Rpl27 vs Hprt*	3.030	0.500		*Hprt vs Gapdh*	− 0.430	0.630		*Ppia vs Rps29*	− 1.060	1.880					
*Rpl27 vs Gapdh*	2.600	0.590		*Hprt vs Rps29*	− 4.570	0.680		*Ppia vs Rpl27*	0.370	2.120					
*Rpl27 vs Rplp0*	0.500	0.730		*Hprt vs Rplp0*	− 2.560	0.910		*Ppia vs Tbp*	5.580	2.400					

Mean ΔCq values are given for the mean difference between the genes. Standard deviations (SD) are given for the variation in Cq values over the animals

### NormFinder analysis

**Fig. 1:**
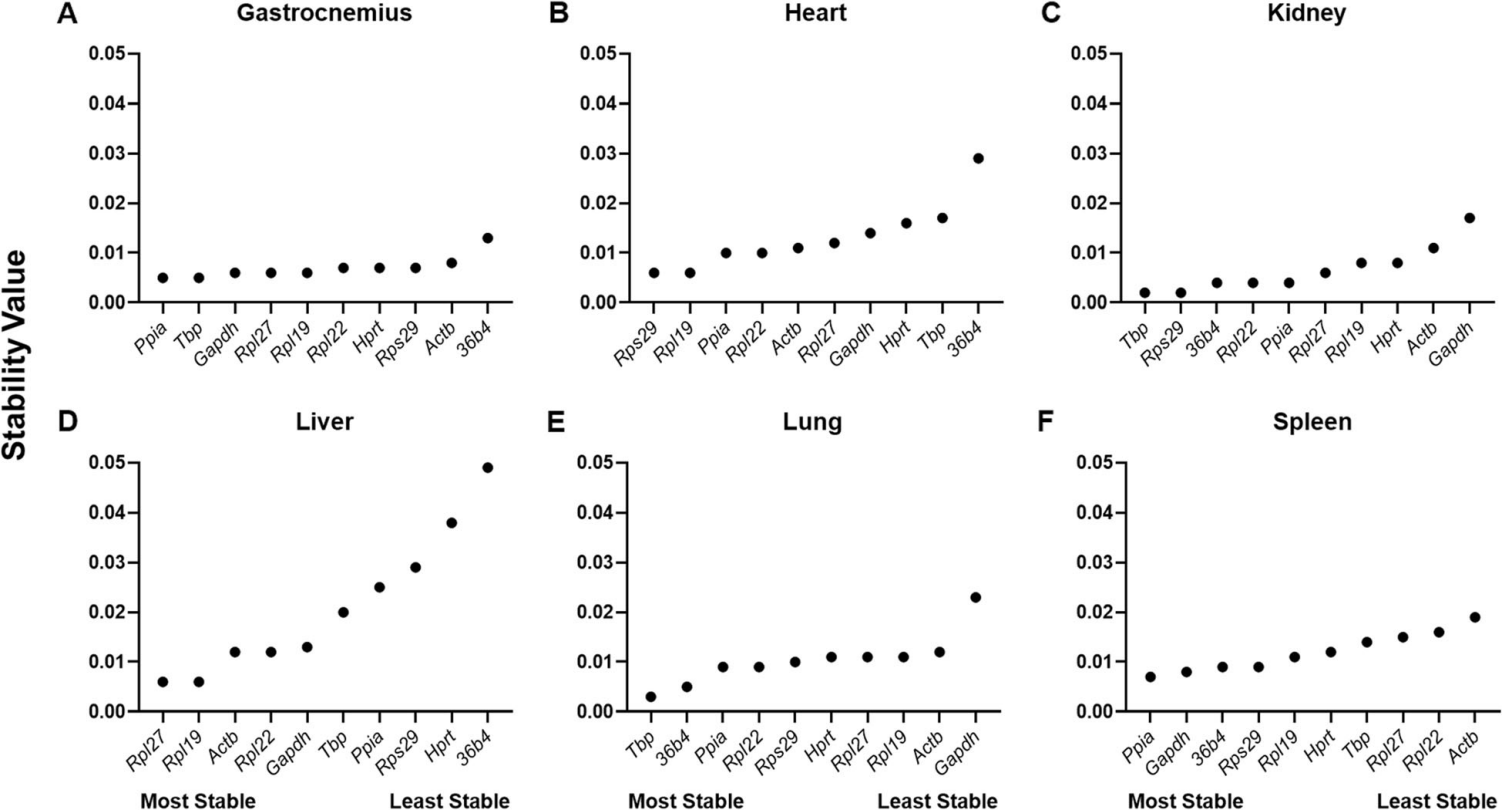
Relative gene stability values of RG including both experimental conditions. Stability values were determined using NormFinder (**A**–**F**). Stability values of RF in the gastrocnemius, heart, kidney, liver, lung, and spleen. Values were plotted based on stability; most stable starting on the left and least stable on the right

**Fig. 2:**
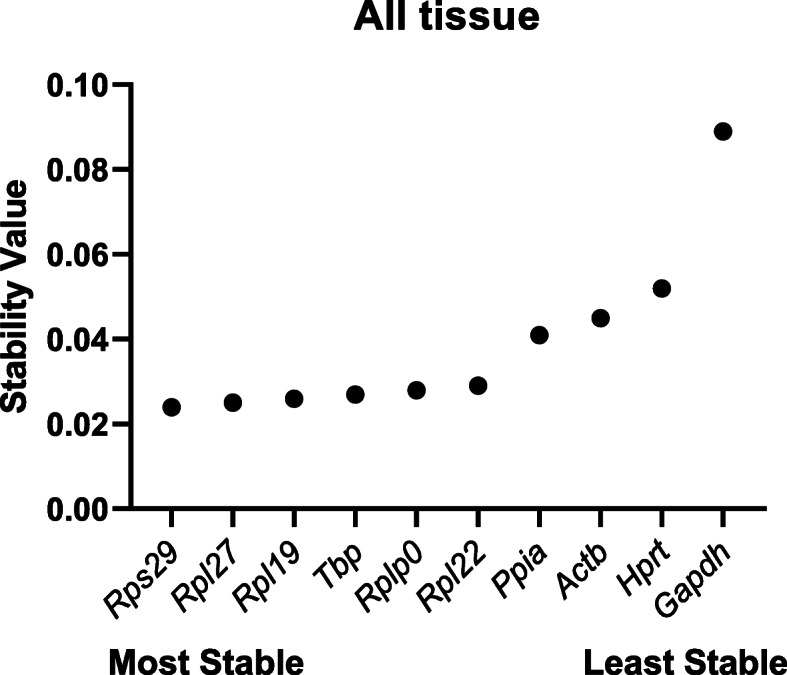
Relative gene stability values of RG. Stability values were determined using NormFinder. Stability values of reference genes based on a combined analysis of gene expression in the gastrocnemius, heart, kidney, liver, lung, and spleen. Values were plotted based on stability; most stable starting on the left and least stable on the right

In contrast to the BestKeeper software, NormFinder determines suitability of RG as a function of variability. NormFinder software ranks potential RG using a model-based approach. The methodology examines sample subgroups (PF and ID herein), disparity in intra- and intergroup expression, and from these data calculates a stability value for candidate RG [2]. RG were assessed first in each tissue individually and then in all tissues combined to determine appropriateness of a single RG for use in all tissues. Among individual tissues, the most stable RG were *Rps29* in the heart, *Tbp* in the kidney and lung, *Rpl27* in the liver, and *Ppia* in the gastrocnemius and spleen. Exhibiting the least stability, *Actb* ranked poorly in nearly all tissues (Fig. 1A–F). After combining data from the six individual tissues, *Rps29* and *Rpl27* were identified as the most stable RG and *Hprt* and *Gapdh* as the least stable RG using NormFinder (Fig. 2).

### RefFinder analysis

**Fig. 3:**
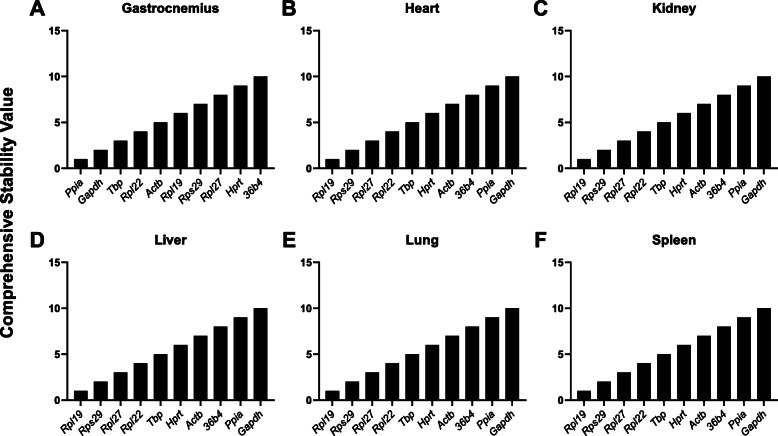
Comprehensive stability ranking of RG including both experimental conditions. Rankings were determined using RefFinder. **A**–**F** Ranking of RG in the gastrocnemius, heart, kidney, liver, lung, and spleen. Values were plotted based on ranking number; most stable [1] and least stable [10]

**Fig. 4:**
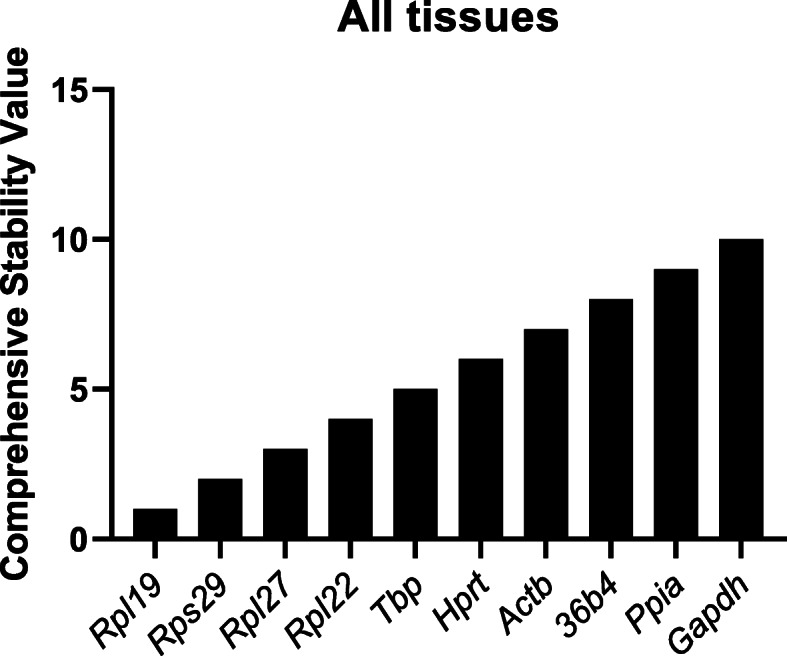
Comprehensive stability ranking of RG including both experimental conditions. Rankings were determined using RefFinder. Ranking of RG was based on a combined analysis of gene expression in the gastrocnemius, heart, kidney, liver, lung, and spleen. Values were plotted based on ranking number; most stable [1] and least stable [10]

RefFinder is a software program that utilizes multiple established algorithms (BestKeeper, ΔCq, geNorm, and NormFinder) to calculate a comprehensive RG stability value [33]. Each gene is assigned a weight based on each algorithm’s geometric mean and weights are then combined to conclude the overall RefFinder ranking. In the individual tissues, the most stable RG were *Hprt* in the heart, *Rps29* in the kidney, *Rplp0* in the lung, *Rpl27* in the liver, and *Ppia* in the gastrocnemius and spleen (Fig. 3A–F). After combining the six tissues, *Rpl19* and *Rps29* were identified as the most stable RG and *Ppia* and *Gapdh* as the least stable (Fig. 4). Interestingly, when experimental conditions were analyzed separately (PF or ID) and combined (PF and ID), *Actb*, *Ppia*, and *Gapdh* all were ranked in the bottom half respectively (data not shown).

Results from BestKeeper, comparative delta Cq, NormFinder, and RefFinder computational programs were organized to develop a relative overall ranking. The ranking was based on PF and ID experimental groups and all tissues combined. The top two candidates (in rank order of most suitable to least suitable) were *Rpl19* and *Rps29*. The least suitable candidate was *Gapdh*, with *Actb*, *Ppia*, and *Rplp0* ranking second in a three-way tie (Table 5)*.* The same analyses were completed for the top two candidates or most suitable genes in each specific tissue (Table 6).

**Table 5:** Relative overall ranking

	Ranking	BestKeeper	ΔCq	NormFinder	RefFinder	Overall
**Most stable**	1	*Hprt*	*Rpl19*	*Rps29*	*Rpl19*	*Rpl19*
	2	*Rpl19*	*Actb*	*Rpl27*	*Rps29*	*Rps29*
**Least stable**	1	*Gapdh*	*Rplp0*	*Gapdh*	*Gapdh*	*Gapdh*
	2	*Actb*	*Ppia*	*Hprt*	*Ppia*	*Ppia*

The two most stable and two least stable RG from BestKeeper, comparative ΔCq, NormFinder, and RefFinder were combined to provide an overall ranking of PF and ID experimental groups in all tissues. Overall ranking was determined by quantifying the most stable or least stable genes of all programs

**Table 6:** Relative overall ranking for each tissue

Gastrocnemius	Ranking	BestKeeper	ΔCq	NormFinder	RefFinder	Overall
**Most stable**	1	*Gapdh*	*Ppia*	*Ppia*	*Ppia*	*Ppia*
	2	*Ppia*	*Rpl22*	*Tbp*	*Gapdh*	*Gapdh*
**Heart**	**Ranking**	**BestKeeper**	**ΔCq**	**NormFinder**	**RefFinder**	**Overall**
**Most stable**	1	*Hprt*	*Rps29*	*Rps29*	*Hprt*	*Rps29*
	2	*Tbp*	*Rpl19*	*Rpl19*	*Rps29*	Rpl19/Hprt*
**Kidney**	**Ranking**	**BestKeeper**	**ΔCq**	**NormFinder**	**RefFinder**	**Overall**
**Most stable**	1	*Gapdh*	*Ppia*	*Tbp*	*Rps29*	*Rps29*
	2	*36b4*	*36b4*	*Rps29*	*Rpl22*	*36b4*
**Liver**	**Ranking**	**BestKeeper**	**ΔCq**	**NormFinder**	**RefFinder**	**Overall**
**Most stable**	1	*Tbp*	*Rpl27*	*Rpl27*	*Rpl27*	*Rpl27*
	2	*Rpl27*	*Rpl19*	*Rpl19*	*Rpl19*	*Rpl19*
**Lung**	**Ranking**	**BestKeeper**	**ΔCq**	**NormFinder**	**RefFinder**	**Overall**
**Most stable**	1	*Gapdh*	*36b4*	*Tbp*	*36b4*	36b4*
	2	*Hprt*	*Tbp*	*36b4*	*Tbp*	Tbp*
**Spleen**	**Ranking**	**BestKeeper**	**ΔCq**	**NormFinder**	**RefFinder**	**Overall**
**Most stable**	1	*36b4*	*Rps29*	*Ppia*	*Ppia*	Gapdh*
	2	*Gapdh*	*Rpl22*	*Gapdh*	*Rps29*	Ppia*
						Rps29*

The two most stable RG from BestKeeper, comparative ΔCq, NormFinder, and RefFinder were combined to provide an overall ranking of PF and ID experimental groups in gastrocnemius, heart, kidney, liver, lung, and spleen. Overall ranking for each tissue was determined by quantifying the most stable genes of all programs. *Indicates equal stability ranking

**Fig. 5:**
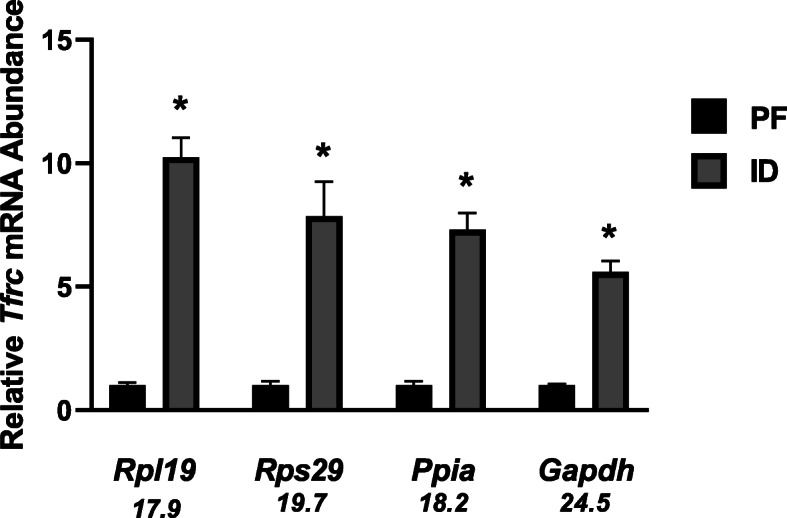
Real-Time quantitative PCR results assessing relative *Tfrc* mRNA expression in the liver. The mRNA levels of *Tfrc1* were normalized to *Rpl19*, *Rps29*, *Ppia*, *and Gapdh* in pair-fed and iron-deficient rat livers

Lastly, to compare the impact of RG on target gene abundance and the interpretation of data, *Tfrc* gene expression in the liver of PF and ID animals was examined. Using the two best RG based on the overall ranking, (*Rpl19* and *Rps29*), and two commonly used genes that ranked poorly in our analyses (*Gapdh* and *Ppia*), the relative abundance of *Tfrc* mRNA was determined using the ddCt method [34]. Although *Tfrc* expression increased significantly in ID animals regardless of the RG utilized, the relative fold changes varied (Fig. 5) (*p* < 0.05). For normalization using *Rpl19* and *Rps29* as RG, *Tfrc* mRNA abundance increased 10-fold and 8-fold in ID animals, respectively (Fig. 5). In contrast to the best ranking RG, using *Gapdh* and *Ppia* as RG, *Tfrc* mRNA abundance increased 6-fold and 7-fold, respectively (Fig. 5).

## Discussion

The necessity for ensuring suitable RG in qPCR quantitation has been well recognized [2, 20, 26, 35, 36]. Ideal RG should exhibit minimal variation in expression levels among various tissues and under experimental manipulations [35]. The existence of an ideal RG is, however, uncertain at best. To date, limited data has been published on gene expression analyses with nutrient-gene interactions in animal models [37, 38] and to our knowledge, an empirical determination of appropriate RG selection in the male weanling rat model of iron deficiency has not been conducted. Additionally, the extent to which RG vary among specific tissues in the same nutritional model has not been examined.

This study was designed to evaluate variation in gene expression in ten commonly used RG in varying dietary (PF and ID) conditions and to identify the RG most suitable for iron deficiency analyses utilizing qPCR in gastrocnemius, heart, kidney, liver, lung, and spleen tissues. Our data is consistent with other research and suggests that commonly used RG may be regulated under experimental conditions and expression stability varies between tissues [26]. It is evident that appropriateness of a RG is likely dependent on the tissue of interest in which gene expression is being analyzed. For example, *Gapdh* ranked poorly in the majority of the computational programs, both in individual tissues and when combining all tissues for analysis. However, NormFinder and RefFinder data concluded *Gapdh* had increased stability and was ranked in the top 3 RG in skeletal muscle. Another example of RG inconsistency based on tissue occurred with *Rplp0*; although *Rplp0* is one of the least stable RG in the liver, it is the most stable RG in the lung based on the RefFinder results. These tissue differences were reflected in poor overall ranking when all tissues were combined for analysis. In contrast, both *Rpl19* and *Rps29* were relatively stable in all tissues resulting in a high overall ranking as determined by all software analyses and our combined overall ranking system. Thus, it is evident that appropriateness of a RG is likely dependent on the tissue of interest in which gene expression is being analyzed, and when comparing multiple tissues simultaneously, it is important RG exhibit relative stability across all tissues.

The RG selected herein have diverse biological functions and origination. The RG can be categorized as encoding for (1) ribosomal proteins, (2) structural proteins, or (3) enzymatic proteins (Table 7). Based on our results, rRNA (*Rpl19 and Rps29*) are the most stable and highest ranking RG for the weanling model of iron deficiency. Although ribosomal RG tend to be more stable in our study, it is important to understand the limitations of using rRNA as RG. First, synthesis of rRNA (RNA polymerase I) and mRNA (RNA polymerase II) are independent, and for that reason, it is thought to be controversial to choose a RG whose transcription is not regulated in the same manner [39]. Second, if original RNA samples were enriched for mRNA, rRNA would be excluded from the isolation process making it an inappropriate control [16]. The samples used in this study were not enriched for any RNA species as evidenced by agarose gel. Next, according to Derveaux et al. [40], it is important to select RG with a similar abundance level to the target mRNA (or gene), making rRNA unsuitable since they are expressed at much higher levels than mRNA. Finally, like mRNA, rRNA have been reported to be regulated under some experimental conditions [20, 41, 42].

**Table 7:** Reference gene information

Gene name	Gene symbol	Accession number	Function
Actin, beta	*Actb*	NM_031144	Cytoskeletal structural protein
Glyceraldehyde-3-phosphate dehydrogenase	*Gapdh*	NM_017008	Glycolysis enzyme
Hypoxanthine phosphoribosyltransferase 1	*Hprt*	NM_012583	Salvages purines
Peptidylprolyl isomerase A (cyclophilin A)	*Ppia*	NM_017101	Protein folding
Ribosomal protein L19	*Rpl19*	NM_031103	Protein synthesis
Ribosomal protein L22	*Rpl22*	NM_031104	Protein synthesis
Ribosomal protein L27	*Rpl27*	NM_022514	Protein synthesis
Ribosomal protein, large, P0 (36b4)	*Rplp0*	NM_022402	Protein synthesis
Ribosomal protein S29	*Rps29*	NM_012876	Protein synthesis
TATA box-binding protein	*Tbp*	NM_001004198	RNA polymerase II transcription factor

The use of computational programs for determination of the most suitable RG assumes consistent gene expression profiles between experimental groups. Our study, consistent with other studies, shows similar results in overall ranking between all computational programs for some genes [43, 44]. This could be due to overlap in computational programs as RefFinder uses a combination of NormFinder, BestKeeper, Delta CT, and Genorm. We did not give more weight to any one program to take an unbiased approach to the analyses. Substantial variation was exhibited by some of the RG under examination. For instance, when analyzing all tissues together in both PF and ID animals, *Actb* ranked as the second most stable gene with the ΔCq method, but then ranked in the bottom half of all genes with BestKeeper, NormFinder, and RefFinder. Thus, this type of result supports a more robust approach to RG selection. Despite some similarities between computational program results, small differences in RG stability do exist and could lead to unreliable data interpretation. For instance, when liver *Tfrc* mRNA abundance levels were normalized to the most stable RG (*Rpl19* and *Rps29*) and the least stable RG (*Actb* and *Gapdh*), as determined by our overall ranking system, *Tfrc* mRNA abundance was significantly increased in the ID animals based on all four RG; however, the magnitude of the differences varied. A significant increase in *Tfrc* mRNA abundance in response to dietary iron deficiency has been well established [45, 46], however in studies aiming to evaluate target mRNA that result in marginal mRNA abundance changes, a significance may not be detected. Therefore, it may be necessary to use multiple computational programs when determining the most stable RG for nutrient-gene interaction-focused studies. Additionally, as suggested by Bustin *et al.* [15], using more than one RG for normalization and choosing the top ranked RG based on the use of multiple computational programs is likely the superior comprehensive approach investigators should use for mRNA normalization.

## Conclusions

Small changes in gene expression may be misinterpreted if an appropriate RG is not selected. Therefore, it may be inappropriate to choose RG for a study based solely on previous research or literature reviews instead of taking an empirical approach to identifying the most suitable RG. To our knowledge, this is the first study to examine RG stability for qPCR gene expression analyses focused on dietary condition and tissue type. Based on the ten-selected RG, *Rpl19 and Rps29* are the most suitable RG for normalization studies involving gastrocnemius, heart, kidney, liver, lung, and spleen tissues in studies focused on the male weanling model of dietary iron deficiency. The combined ranking system provides a more appropriate evaluation of RG suitability because it provides a thorough assessment of overall RG stability based on four accepted computational RG programs. The model illustrated herein provides an appropriate method for validation of RG, specifically for studies involving dietary responses in multiple tissues, and should be implemented prior to qPCR assays in order to report valid and reliable results.

## Methods

Twenty-one-day-old weanling male Sprague-Dawley® (Harlan, Indianapolis, IN USA) rats (*n* = 24) were housed individually in stainless steel, wire-bottomed cages in an environmentally controlled facility and maintained on a 12-h light: dark cycle at 20 °C with ad libitum access to deionized water. All rats were allowed access to the control diet for 3 days prior to starting dietary treatments. Following the acclimation period, animals were randomly assigned to one of three diet groups (*n* = 8 per group) for 21 days: control iron-replete (C; 40 mg Fe/kg diet), pair-fed iron-replete (PF; control diet with the mean intake of the ID group) or iron-deficient (ID; < 5 mg Fe/kg diet). Diets were purchased from Harlan Teklad (ENVIGO, Madison, WI, USA; C-TD.89300 and ID-TD.80396) based on the recommendations from the American Institute of Nutrition’s 1976 (AIN 76) Standards for Nutritional Studies. Individual body weights and food intake were measured daily. Following the 21-day experimental period, animals were anesthetized with a ketamine/xylazine mixture and were sacrificed by exsanguination via the abdominal aorta. The gastrocnemius, heart, kidney, liver, lung, and spleen were snap-frozen in liquid nitrogen immediately following removal and stored at – 80 °C until subsequent analysis. All institutional guidelines for the care and use of laboratory animals were followed and approved by the OSU Institutional Animal Care and Use Committee.

### RNA isolation and cDNA synthesis

Total RNA was isolated from tissues including the gastrocnemius, heart, kidney, liver, lung, and spleen using STAT-60 (Tel-test, Inc., Friendswood, TX, USA) according to the manufacturer’s instructions. After isolation, RNA concentration was determined using a Nanodrop spectrophotometer (Thermo Fisher Scientific, Waltham, MA, USA) and relative purity of total RNA was assessed by the A_260/280_ ratio. Only A_260/280_ ratios ≥ 1.8 were used for this study. The integrity of RNA was determined by examining 18S and 28S rRNA by agarose gel electrophoresis. Total RNA was treated with DNase I (Roche, Basel, Switzerland) and reverse-transcribed with SuperScript II (Invitrogen, Carlsbad, CA, USA) for a final cDNA concentration of 50 ng/μL.

### Quantitative qPCR and data analysis

Gene expression was determined by qPCR using SYBR Green chemistry on an ABI 7900HT sequence detection system instrument and 2.4 SDS software (Applied Biosystems, Foster City, CA, USA). All reactions were performed in 10 μL volumes, including 50 ng of template, 2.5 μM of each forward and reverse primer, and 10 mM of dNTPs (2.5 mM each). Amplification was performed with a 2 min activation step at 50 °C, 10 min denaturation step at 95 °C, followed by 40 cycles of 90 °C for 15 s and 60 °C for 1 min. Following each cycle, a dissociation curve analysis was performed using the default settings of the software to confirm the specificity of the PCR products. For each target RG, the relative stability was assessed using BestKeeper, the comparative delta Cq (ΔCq) method, NormFinder, and RefFinder.

RG were assessed in individual tissues and based on all tissues combined. They were assessed between experimental conditions (PF and ID) based on all tissues combined. Potential RG analyzed included *Actb*, *Gapdh*, *Hprt*, *Ppia*, *Rpl19*, *Rpl22*, *Rpl27*, *Rplp0*, *Rps29*, and *Tbp* (Table 7). The overall rankings were determined by using all tissue combined analyses or each specific tissue for each stability program. The two most stable and two least stable RG for each program were quantified based on the number of times they were ranked at the top or bottom. The two most stable and two least stable genes from the all tissues ranking were further used as RG to compare *Tfrc* gene expression in PF versus ID rat livers. The comparative ΔΔCq method was used to analyze mRNA abundance [47]. Oligonucleotide primers (Table 8) were obtained from Integrated DNA Technologies (IDT, Coralville, IA, USA) and designed using Primer Express software 3.0.1 (Applied Biosystems, Foster City, CA, USA). Briefly, nucleotide sequences were acquired from NCBI and primers were designed to cross exons, not exceed an amplicon length of 100 nucleotides, and have the lowest possible error rate.

**Table 8:** Primer sequences for reference gene analysis by qPCR

Gene symbol	Forward primer	Reverse primer
*Actb*	5′ CAT CGT GGG CCG CCC TA	5′ CGC CCA CGG AGG AGT CCT TCT G
*Gapdh*	5′ GAG GTG ACC GCA TCT TCT TG	5′ CCG ACC TTC ACC ATC TTG TC
*Hprt*	5′ GCC GAC CGG TTC TGT CAT	5′ CAT AAC CTG GTT CAT CAT CAC TAA TCA
*Ppia*	5′ GGT CTT TGG GAA GGT GAA AGA A	5′ GCC ATT CCT GGA CCC AAA A
*Rpl19*	5′ CGT CCT CCG CTG TGG TAA A	5′ TGG CGA TTT CGT TGG TTT
*Rpl22*	5' CAC CCT GTA GAA GAT GGA ATC ATG	5' TTC CCG TTC ACC TTG ATC CT
*Rpl27*	5′ GCA AAG CCG TCA TCG TAA AGA	5′ CTG GGA TAG CGG TCA ATT CC
*Rplp0*	5′ CAC CTT CCC ACT GGC TGA A	5′ TCC TCC GAC TCT TCC TTT GC
*Rps29*	5′ GCC AGG GTT CTC GCT CTT G	5′ GGC ACA TGT TCA GCC CGT AT
*Tbp*	5′ TGC CAG AAA TGC TGA ATA TAA TCC	5′ GTT CGT GGC TCT CTT ATT CTC ATG
*Tfrc*	5′ TCG GCT ACC TGG GCT ATT GT	5′ CCG CCT CTT CCG CTT CA

### Statistical analysis

Analyses were performed using SPSS version 23.0 software (IBM Corp., Armonk, NY, USA). Statistical analyses using Student’s *t* test were performed to determine treatment effects of dietary condition (ID or PF). Values are expressed as means ± standard error of the mean (SEM).

## Data Availability

The datasets used and/or analyzed during the current study are available from the corresponding author on reasonable request.

## Abbreviations

Actb: Actin, beta
C: Control
CP: Crossing points
∆Cq: Comparative delta quantification cycle
Gapdh: Glyceraldehyde-3-phosphate dehydrogenase
Hprt: Hypoxanthine phosphoribosyltransferase 1
ID: Iron deficiency
IRP: Iron regulatory protein
PF: Pair-fed
Ppia: Peptidylprolyl isomerase A
qPCR: Quantitative real-time polymerase chain reaction
RG: Reference genes
Rpl19: Ribosomal protein L19
Rpl22: Ribosomal protein L22
Rpl27: Ribosomal protein L27
36b4: Ribosomal protein large P0
Rps29: Ribosomal protein S29
SDCq: Standard deviation crossing point
Tbp: TATA box-binding protein
Tfrc: Transferrin receptor
